# Self-care for common colds: A European multicenter survey on the role of subjective discomfort and knowledge about the self-limited course - The COCO study

**DOI:** 10.1371/journal.pone.0195564

**Published:** 2018-04-13

**Authors:** Anika Thielmann, Biljana Gerasimovska-Kitanovska, Tuomas H. Koskela, Vildan Mevsim, Birgitta Weltermann, for the European General Practice Research Network Working Group on Self-Care

**Affiliations:** 1 Institute for General Medicine, University Hospital Essen, University of Duisburg-Essen, Essen, Germany; 2 Department of Family Medicine/Department of Nephrology, University St. Cyril and Methodius, Skopje, Macedonia; 3 University of Tampere, Department of General Practice, Tampere, Finland; 4 Department of Family Medicine, Dokuz Eylul University, Faculty of Medicine, Izmir, Turkey; 5 Institute for General Practice and Family Medicine, University Hospital Bonn, University of Bonn, Bonn, Germany; Veterans Affairs New Jersey Healthcare System, UNITED STATES

## Abstract

**Introduction:**

Common colds are the most frequently encountered disease worldwide and the most frequent reason for self-care. According to the cross-sectional European Common Colds study (COCO), patients use as many as 12 items on average for self-care. Little is known about the influence of discomfort and knowledge on self-care for common colds.

**Main objective:**

To understand the influence of patients’ discomfort during a cold and their knowledge about the self-limited disease course on the use of self-care measures.

**Materials and methods:**

This COCO analysis included 2,204 patients from 22 European primary care sites in 12 countries. Each site surveyed 120 consecutive adults with a 27-item questionnaire asking about patients’ self-care, subjective discomfort during a cold (discomfort: yes/no), and knowledge about the self-limited course (yes/no). Country-specific medians of the number of self-care items served as a cut-off to define high and low self-care use. Four groups were stratified based on discomfort (yes/no) and knowledge (yes/no).

**Results:**

Participants’ mean age was 46.5 years, 61.7% were female; 36.3% lacked knowledge; 70.6% reported discomfort. The group *has discomfort/no knowledge* exhibited the highest mean item use (13.3), followed by *has discomfort/has knowledge* (11.9), *no discomfort/no knowledge* (11.1), and *no discomfort/has knowledge* (8.8). High use was associated with discomfort (OR 1.8; CI 1.5–2.2), female gender (OR 1.7; 1.4–2.0), chronic pain/arthritis (OR 1.6; 1.2–2.1), more years of education (OR 1.3; 1.1–1.6), age <48 years (OR 1.3; 1.0–1.5), and lack of knowledge (OR 1.2; 1.0–1.4).

**Discussion:**

Counseling on common colds should address patients’ discomfort and soothing measures in addition to providing information on the natural disease course.

## Introduction

The common cold is the most frequently encountered human disease worldwide [1]. The incidence is subject to seasonal variation (more episodes in winter and fall) [1] and is known to be age-specific [2], with 6-8 episodes yearly in younger children, decreasing to 2-4 episodes in adults [3]. According to a US study (2015) with 3,333 participants, 85% of all adults will develop at least one common cold per year lasting between three and seven days [4]. Common colds have a tremendous economic burden on societies due to absenteeism at school [5] and at work. For the US, a loss of 70 million workdays annually was shown, corresponding to US$8 billion in indirect costs [6, 7]. Despite this tremendous impact on society, medical literature addresses common colds rather poorly, mainly because common colds are considered minor illnesses with a self-limited course.

For common colds, patients typically seek medical counseling for reassurance and sick leave and are advised to use soothing measures [8, 9]. Our Common Colds study (the COCO study) with 2,724 patients from 22 primary care sites in 12 European countries identified a total of 527 self-care practices for common colds. The age-standardized mean item use per participant was 12 (range 6 to 15), and the majority of patients (62%) used a combination of pharmaceutical and non-pharmaceutical products (see [10]). The COCO study was initiated because unsystematic observations by general practitioner (GP) researchers from the working group had indicated that patients use a multitude of measures for colds, but no details were available at the time.

The aim of this publication is to better understand factors driving this self-care behavior, especially the influence of knowledge and discomfort on the number of measures used. In behavioral sciences, the knowledge about the disease and subjective suffering (discomfort) are factors known to influence health-care behaviors. Aiming to support evidence-based self-care, a better understanding of the role of knowledge on the self-limited disease course and the subjective discomfort is considered important for patient education in physician offices as well as public campaigns.

This analysis of the COCO data aims at understanding the influence of patients’ discomfort during a cold and their knowledge about the self-limited course of the disease on the use of self-care.

## Methods and materials

### Study design and population

The COCO study is a cross-sectional multicenter questionnaire survey designed and conducted by the Working Group on Self-Care for Common Colds of the European General Practice Research Network (EGPRN). The Ethics Committee of the Faculty of Medicine at the University of Duisburg-Essen, Germany, provided the first ethical approval (13-5495-BO) which was subsequently used by all study site coordinators to obtain additional ethical approval according to regional requirements. Participants were informed about their anonymous and voluntary participation. Completion of the questionnaire was regarded as an indication of participants’ consent to the study. Details of the rationale of the COCO study design have been published elsewhere [11]. Briefly, in order to ensure a random sample, 120 consecutive patients aged ≥18 years visiting their general practitioner for any reason in fall/winter 2013/14 were asked to participate. Patients were asked to answer the following question: ‘When you had a common cold during the last year: what did you do?’ A total of 3,074 questionnaires were returned from 27 sites in 14 European countries. After excluding data from sites with different sampling strategies (physician interviews, older questionnaire version, incomplete questionnaire distribution) the study population available for analysis comprised 2,724 patients.

### Study instrument

The patient questionnaire was developed based on a survey among 10 primary care physicians from seven European countries (Austria, Bosnia and Herzegovina, Germany, Israel, Italy, Macedonia, and Poland) and one EU-associated country (Turkey). Physicians were asked to list typical self-care items used by their patients for common colds [11]. The final patient questionnaire consisted of 27 questions which included 94 self-care practices plus free text options in 11 categories: over-the-counter medication (11 items), specific foods or drinks (11 items), herbal teas (18 items), alcoholic drinks (3 items), self-prepared special recipes (7 items), pastilles or drops (10 items), something for the nose (4 items), inhalations (8 items), gargle or sprays for the throat (4 items), items applied externally (5 items), and extras at home (13 items). Additionally, various patient characteristics were requested, e.g. age, gender, health insurance status, number of school years, number of pills taken daily, and frequent chronic conditions (depression, chronic kidney disease, chronic pain/arthritis/osteoarthritis, asthma/chronic bronchitis, diabetes, heart disease, high blood pressure). Subjective discomfort was measured by asking whether the patient ‘felt very poorly during the last common cold’ (answer options: yes, no, don’t know). Knowledge on the self-limited nature of the disease was assessed by asking whether ‘a common cold goes away by itself’ (answer options: yes, no, don’t know).

### Classification of items

Two researchers classified all single self-care items by their mode of application: ‘intestinal absorption’, ‘intranasal application’, ‘local oral effects’, ‘inhalation’, ‘topical use in throat’, ‘external use’, ‘foodstuffs’, and ‘extras at home’. All pharmacological items were further sub-classified using the international ATC (WHO Anatomical Therapeutic Chemical) classification system for pharmacological substances, which divides active substances into groups according to the organ or system on which they act as well as their therapeutic, pharmacological and chemical properties [12]. Items not listed were assorted in plausible groups and sub-categories based on content (e.g., ‘teas’, ‘fruit’, ‘vegetables’, ‘lozenges’ or ‘alcohol’): in this sub-classification, items were listed individually if they had a utilization rate of at least 1% (n ≥ 27) of the sample; otherwise they were summarized as ‘other’ within each mode. All items with an assigned ATC code and the unspecific answer options ‘antibiotics’ and ‘pain medication’ were called ‘pharmaceutical products’, all other items were called ‘non-pharmaceutical products’. Disagreement between researchers was solved by discussion until a consensus was reached.

### Statistical analysis

Descriptive analyses were performed for the total study population and stratified by country. The response rate was calculated per site and country. For this analysis, percentages reported for ‘knowledge on the self-limited course’ (yes/no) and ‘subjective discomfort’ (yes/no) were stratified by country and site.

Four groups were stratified based on ‘knowledge’ yes/no and ‘discomfort’ yes/no (note: ‘yes’ indicated as ‘+’ and ‘no’ as ‘-’): group 1: +knowledge/-discomfort; group 2: -knowledge/-discomfort; group 3: +knowledge/+discomfort; group 4: -knowledge/+discomfort. We hypothesized that groups 1 and 4 would show the largest contrast, with group 1 having the most beneficial characteristics in terms of knowledge and discomfort. Groups 2 and 3 were considered intermediate groups. Analyses for the four groups included: a) the mean total item use and standard deviation; b) the mean item use for the categories non-pharmaceutical/pharmaceutical products and self-care with/without proof of evidence; c) descriptions of the modes of application calculated as the percentage of patients using at least one item per mode, and the mean item use for each mode; d) depending on the properties of the scales, either χ^2^-tests for categorical data or ANOVA and non-parametric tests (Kruskall-Wallis) for continuous data were used to compare groups.

In order to identify factors associated with using more self-care items than 50% of the respective country sample, multivariate logistic regressions were performed. Independent determinants were discomfort (yes), knowledge (no), gender (female), age (48), years of education (above country-specific median), chronic condition(s) (yes), daily pill intake (yes), and smoking status (yes). It was tested for collinearity (Pearson’s r), and determinants significant in bivariate analyses were included in the multivariate regression model. To prevent the multiple inclusion of the chronic disease state in the multivariate regression analysis, only chronic pain/arthritis/osteoarthritis was included. This condition was chosen because these patients typically have analgesics available for regular or occasional use. Their use of self-care including analgesics is likely to be higher than that of participants without access to this medication. The nominal statistical significance level was p<0.05. To correct for multiple testing, the Benjamini-Hochberg procedure was applied [13]. All statistical analyses were performed using IBM® SPSS® Statistics, version 20.

## Results

### Characteristics of the participating countries, sites and patients

A total of 2,724 patients were available for data analysis. The response rate differed between countries, ranging from 53.3% to 89.7%. These numbers are not precise estimates as some sites diverted from the sampling design (the response rate for Macedonia could not be calculated). See S1 Table for the number of participants included per site.

222 patients with missing data for knowledge and/or discomfort and 298 patients who answered ‘don’t know’ for knowledge and/or discomfort were excluded. The final analysis included 2,204 patients (mean age of 46.5±16.41 years, 62.5% female, 12.8±4.46 years of education). Excluded patients did not differ significantly from the final sample with regard to age, gender, years of school, daily pill intake, and chronic conditions (depression, chronic kidney disease, chronic pain/arthritis/osteoarthritis, asthma/chronic bronchitis, diabetes, heart disease, high blood pressure). See Table 1 for baseline characteristics; site characteristics have been published elsewhere (see [10]).

**Table 1:** Characteristics of the COCO study sites and participants (n = 2,204).

		n	%
Gender, female		1,360	62.5
Mean age (SD)		46.5 (16.4)	
Insurance status, public		2,052	96.4
Non-smoker		1,683	77.8
Patients with ≥1 self-reported chronic condition:		871	39.5
	Hypertension	488	56.0
	Chronic pain/arthritis/osteoarthritis	215	24.7
	Heart disease	192	22.0
	Diabetes	170	19.5
	Asthma/chronic bronchitis	151	17.3
	Depression	117	13.4
	Chronic kidney disease	44	5.1
Number of tablets used daily (mean, SD)		2.0	2.76
	Patients with ≥1 tablets daily (n = 1,204):		
	ASA*/aspirin	256	21.3
	Oral contraceptive	134	11.1
	Anticoagulants	56	4.7
Austria°		86	3.9
Finland		77	3.5
France, 3 sites		265	12.0
Germany, 3 sites		292	13.2
Israel^+^		104	4.7
Italy, 2 sites		148	6.7
Macedonia°		269	12.2
Poland, 2 sites		195	8.8
Slovenia°		89	4.0
Spain^+^		64	2.9
Sweden^+^		77	3.5
Turkey, 5 sites		538	24.4

*ASA = acetylsalicylic acid

°mixed (urban/rural)

^+^urban

### Knowledge of the self-limited course and subjective discomfort during a common cold

One third (36.3%) of the participants did not know about the disease’s self-limited course, with a variation of 6.5% to 60.5% between countries and sites from the same countries. More than two thirds of the participants (70.6%) reported high discomfort during their last common cold, with a range of 40.3% to 90.3% between countries and sites. See additional file 1 in the Supplementary Material for characteristics per data sampling site/primary care practice.

3.3. Self-Care Stratified by Knowledge and Discomfort (n = 2,204)

Stratified by knowledge/discomfort, the size of the four groups was as follows: group 1 (no discomfort/has knowledge) consisted of 23.1% of the participants, group 2 (no knowledge, no discomfort) of 6.3%, group 3 (has discomfort/has knowledge) of 40.6%, and group 4 (has discomfort/no knowledge) consisted of 30.0%.

The mean item use increased with increasing group number, ranging from 8.8 to 13.3. In general, patients with discomfort (groups 3 and 4) used more self-care measures than those in groups 1 and 2. The largest differences for self-care were identified when comparing groups 1 (no discomfort/has knowledge) and 4 (has discomfort/no knowledge). For five of eight application modes, patients in group 4 (has discomfort/no knowledge) reported a higher use than those in group 3 (has discomfort/has knowledge). Across the four groups, patients used about 9.1 more non-pharmaceutical than pharmaceutical products. Interestingly, group 3 used the highest number of pharmaceuticals. Patients in group 4 (has discomfort/no knowledge) used 53% more non-pharmaceutical products than those in group 1 (no discomfort/has knowledge). See Table 2 for details.

**Table 2:** Self-care behavior stratified by four patient groups based on the presence and/or absence of knowledge about the self-limited disease course and subjective discomfort during the last common cold (n = 2,204).

	Group 1 ☺		Group 2		Group 3		Group 4 ☹		p-value
	(+) knowledge (-) discomfort		(-) knowledge (-) discomfort		(+) knowledge (+) discomfort		(-) knowledge (+) discomfort		
**Group size, n, %**	510	23.1	138	6.3	894	40.6	662	30.0	
**Group characteristics**									
Age, mean	48.4±17.29		48.7±16.41		46.1±16.27		45.2±15.75		<0.01
Gender: female, %	54.9		56.0		63.7		68.1		<0.001*
Years of school (including higher education), mean	13.3±4.16		10.7±4.82		13.3±4.39		12.3±4.52		<0.001*
**Mean total use**	8.8±5.50		11.1±6.30		11.9±6.45		13.3±7.39		<0.001*
**Spectrum on mode of application level: at least 1 in %**									
	n	%	n	%	n	%	n	%	
Foodstuffs	465	**91.2**	131	**94.9**	860	**96.2**	643	**97.1**	<0.001*
Intestinal absorption	358	**70.2**	97	**70.3**	775	**86.7**	543	**82.0**	<0.001*
Extras at home	48	**65.5**	15	**74.6**	107	**85.3**	114	**87.0**	<0.001*
Intranasal use	235	**46.1**	58	**42.0**	521	**58.3**	350	**52.9**	<0.001*
Inhalation	155	**30.4**	57	**41.3**	321	**35.9**	261	**39.4**	<0.01
Local oral effects	138	**27.1**	48	**34.8**	339	**37.9**	247	**37.3**	<0.001*
Topical use in throat	124	**24.3**	50	**36.2**	289	**32.3**	282	**42.6**	<0.001*
External use	334	**9.4**	103	**10.9**	763	**12.0**	576	**17.2**	<0.001*
**Mean use of items per mode of application group**									
Foodstuffs	4.2±3.25		5.7±3.64		5.1±3.54		6.1±3.99		<0.001*
Intestinal absorption	1.4±1.30		1.5±1.47		2.1±1.59		2.1±1.74		<0.001*
Extras at home	1.5±1.55		1.9±1.71		2.3±1.80		2.5±1.85		<0.001*
Intranasal use	0.5±0.66		0.5±0.65		0.7±0.74		0.6±0.69		<0.001*
Inhalation^#^	0.5±0.78		0.6±0.78		0.6±0.93		0.6±1.03		0.011
Local oral effects^#^	0.4±0.69		0.5±0.75		0.6±0.88		0.5±0.81		<0.001*
Topical use in throat^#^	0.3±0.52		0.4±0.57		0.4±0.56		0.5±0.65		<0.001*
External use^#^	0.1±0.36		0.1±0.34		0.1±0.41		0.2±0.48		<0.001*
**Mean use of pharmaceutical vs. non-pharmaceutical products**									
Non-pharmaceutical products	8.1±5.36		10.5±6.09		10.9±6.29		12.4±7.06		<0.001*
Pharmaceutical products	0.7±0.78		0.6±0.77		1.0±0.87		0.8±0.88		<0.001*
**Ratio pharmaceuticals /non-pharmaceuticals**^#^	1:6.7		1:10.0		1:9.1		1:10.1		<0.001*
**Mean use of self-care with evidence base vs. self-care without evidence base**									
Without evidence base	7.9±5.3		10.4±6.1		10.7±6.3		12.2±7.0		<0.001*
With evidence base	0.9±0.9		0.7±0.9		1.3±1.0		1.0±1.0		<0.001*
**Ratio pharmaceuticals /non-pharmaceuticals**^#^	1:6.1		1:9.6		1:7.8		1:9.6		<0.001*

^#^Kruskal-Wallis test

*Significant after correcting for multiple testing using the Benjamini & Hochberg procedure

### Factors associated with higher use of self-care practices

**Fig 1:**
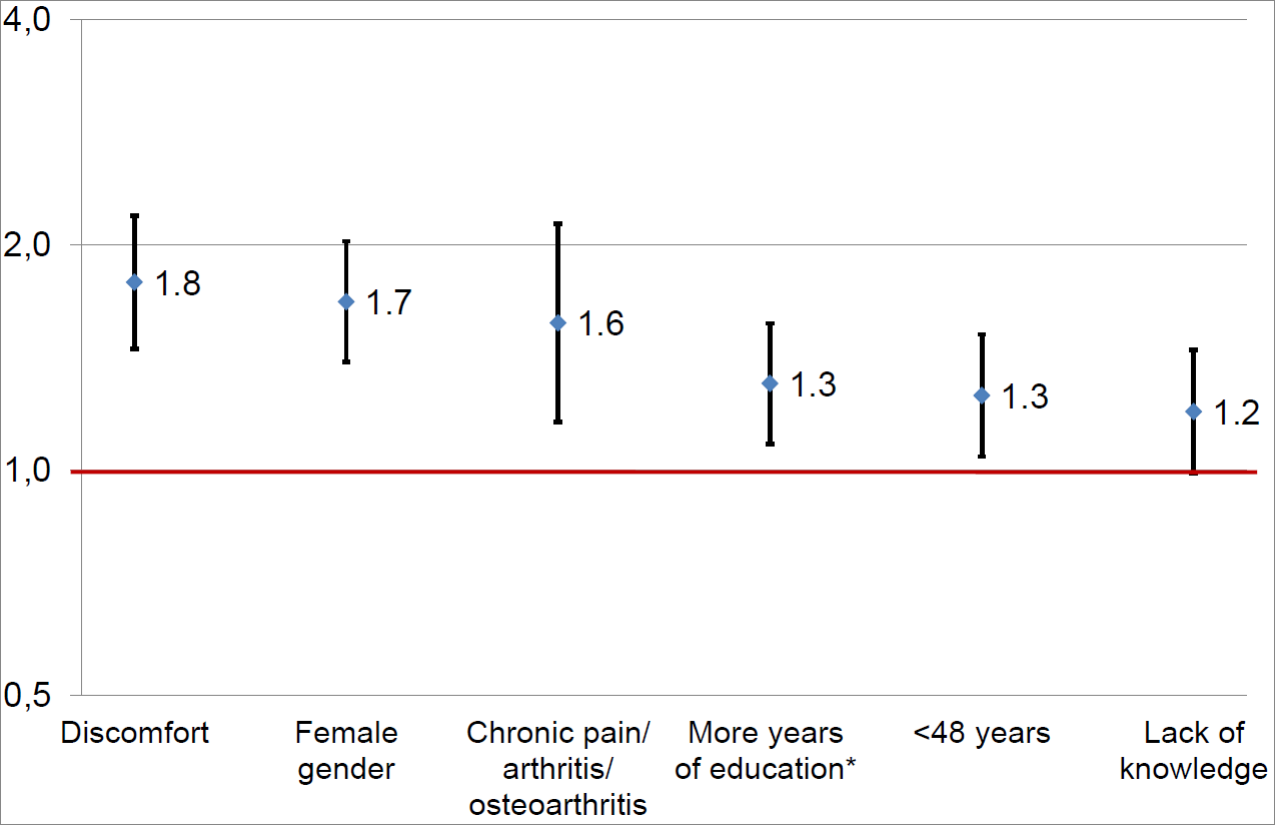
Logistic regression analysis for high self-care use (n = 2,084).

Bivariate analyses showed that a higher number of self-care items used was associated with lack of knowledge (33.2% vs. 40.1%, p = 0.001), having discomfort (64.4% vs. 78.3%, p<0.001), female gender (56.7% vs. 69.8%, p<0.001), younger age (51.4% vs. 58.4%, p = 0.001), more years of education (39.6% vs. 45.9%, p = 0.003), chronic pain/arthritis/osteoarthritis (8.4% vs. 11.4%, p = 0.017), high blood pressure (23.8% vs. 20.1%, p = 0.041; n.s. after correcting for multiple testing), diabetes (8.7% vs. 6.4%; p = 0.044 n.s. after correcting for multiple testing), and depression (4.2% vs. 6.6%, p = 0.013). Only chronic pain/arthritis/osteoarthritis was included. The multivariate regression analysis (Fig 1) showed that high self-care use was most strongly associated with having discomfort (OR 1.8, CI 1.5–2.2), followed by female gender (OR 1.7, CI 1.4–2.0), chronic pain/arthritis/osteoarthritis (OR 1.6, CI 1.2–2.1), more years of education (OR 1.3, CI 1.1–1.6), younger age (below 48 years) (OR 1.3, CI 1.0–1.5), and lack of knowledge (OR 1.2, CI 1.0–1.5) (Nagelkerke R2 = 0.66). A sub-analysis excluding patients with chronic conditions yielded comparable estimates. Regression analyses showed similar estimates for discomfort (OR 2.0, CI 1.7–2.4) and knowledge (OR 1.4, CI 1.1–1.6).

## Discussion

This European study on self-care for common colds showed that having discomfort during colds was frequent among patients (71%), and that 41% of patients also lacked knowledge on the self-limited course. In agreement with our hypothesis, individuals with discomfort who lacked knowledge showed the highest use of self-care. In contrast, participants with knowledge lacking discomfort used 50% fewer items on average. Also, we observed a gradient in self-care over the four stratified groups. Our findings suggest that subjective discomfort is a key factor driving self-care behavior for common colds and needs to be adequately addressed in physician-patient consultations alongside providing information on the natural disease course.

### Comparison with other studies

The literature suggests that self-care behavior is illness-specific, e.g. differing between self-care for upset stomach and bowel irregularity [14]. Therefore, caution is needed when comparing our results to self-care for other illnesses. Surprisingly few studies are available for comparison, although common colds are the most frequent disease and are associated with tremendous socio-economic costs. Although several studies report that symptoms of common colds are the most frequent reason for self-care [14–17], the illness-specific literature on self-care targets predominantly chronic rather than acute, self-limited conditions.

Our approach of distinguishing four groups based on the characteristics discomfort and knowledge is a novelty in the field of self-care for acute diseases. However, illness perception, namely patients’ subjective beliefs and emotional responses to their illnesses, are known to influence patients’ coping, self-management, and thereby outcomes for chronic diseases, e.g. in asthma [18]. Our finding that subjective discomfort influences self-care corresponds to a nationwide survey on self-medication in the German population: the key motive for self-medication (88.3% of the respondents) is patients’ perception of the severity and the assessment that minor health conditions do not require immediate physician consultation [16]. Similarly, a recent Japanese study showed that the health-related quality of life was significantly higher among patients with colds relying on self-medication first compared to patients who visited a physician immediately [19], although further research is needed to better understand the cause-effect relationship.

We identified no study comparable to COCO that assessed knowledge of the self-limited course. However, other studies addressing knowledge and/or misconceptions about the effectiveness of various measures used for colds are well described in the British general population, and showed that up to 35% of participants had misconceptions about the effectiveness of garlic, chicken soup and antibiotics, and 53.2% did not know that viruses caused colds [20]. Addressing reasons for physician consultations, the study showed that neither knowledge about the effectiveness of various measures nor age or education were significant, while there was a positive association for a physician consultation-prone attitude (OR 3.6, p<0.01) and a poorer self-rated health status (OR 1.5, p<0.05).The level of discomfort, however, was not addressed.

Our finding that the current health status (here: the presence of chronic pain and/or arthritis and/or osteoarthritis) is associated with higher self-care use is in agreement with a Spanish National Health Survey with 20,311 participants of the general population, which showed that the prevalence of self-medication was greater among those with a chronic illness (PR 1.2, CI 1.1–1.3) [21]. A Canadian study on self-medication specifically for upset stomach also reported a higher use in participants with a generally poor health status [14]. However, the association between health status and self-care was not consistent across studies. For example, the same Canadian study failed to show a significant association between health status and self-medication of bowel irregularity [14], as did a longitudinal study in Australians (age 65+) separately for the use of self-medication and complementary alternative medicine in general [22]. Neither of the studies addressed discomfort and knowledge, which might explain these heterogeneous results.

Similarly, our result that females, patients with more years of education, and those of younger age exhibit a higher use of self-care for various conditions is not consistently supported across studies. Prior studies addressing self-care in general reported that females use more self-medication than males in Spain (PR 1.21, 95% CI 1.10–1.33) [21], use more home remedies in Canada [14], use a larger variety of home remedies in Germany [15], and are more regular users of over-the-counter (non-prescription) analgesics in Northern Ireland (48% vs. 40.5%, p<0.01) [23]. However, no association between gender and over-the-counter use was found in a random sample of the German general population [16] and in elderly Australians [22]. Our association between younger age and higher self-care use is in line with an Australian and a Japanese survey [19, 24]. The latter considers a better physical condition and less fear of a more serious illness as explanations of why younger patients are more likely to rely on self-care before consulting a physician [19]. In accordance with our finding, the Spanish national survey [21] identified an association of university-level education with the use of self-care (PR 1.25, 95% CI 1.03–1.53). In contrast, Canadians with less formal education were more likely to use self-medication for an upset stomach and bowel irregularity [14]. To our surprise, years of education and knowledge of the self-limited course of common colds were not correlated in our study. And, even more interesting, those with better knowledge and discomfort were more likely to use pharmaceuticals than those who did not know about the disease’s natural course, although non-pharmaceuticals were used about six to ten times more frequently than pharmaceuticals in all four groups.

### Strengths and limitations of the study

This is the first study to analyze self-care for common colds on a European scale. The strength of our study is that it differentiates the influence of discomfort and knowledge on self-care for common colds using four patient groups. However, knowledge was assessed with only one question and necessitates further research. As this study was not designed to provide country-representative results, we abstained from country-specific comparisons. In addition, participants were not asked for their intention to use certain practices and their appraisal of the evidence for various self-care practices. Also, a recall bias may apply as participants were asked about their self-care behavior during their last common cold irrespective of the reason for their practice visit.

## Conclusions

Our study suggests that the mere knowledge that common colds are self-limited is of limited value for patients who experience a high level of discomfort. This finding is important for physician consultations and the design of public health interventions which would act wisely to focus on relieving measures for patients’ discomfort in combination with providing information on the natural disease course. Given that the mere distribution of written patient information has little effect [25, 26], future research needs to identify the complexity of interventions necessary to promote a rational use of evidence-based self-care for common colds.

## Supporting information

S1 Table Appendix. Knowledge about the self-limited disease course and subjective discomfort during the last common cold stratified by country and site (n = 2,204).
(DOCX)

## Abbreviations

ASA: acetylsalicylic acid
ATC code: Anatomical Therapeutic Chemical code
CI: Confidence Interval
COCO: Common Colds study
EGPRN: European General Practice Research Network
N.S.: not significant
OR: Odds Ratio
PR: Prevalence Ratio
SD: Standard Deviation
Vs.: versus
WHO: World Health Organization
