# Supplementary material for: Self-care for common colds: A European multicenter survey on the role of subjective discomfort and knowledge about the self-limited course - The COCO study
Source: PLoS One. 2018 Apr 13;13(4):e0195564. doi: 10.1371/journal.pone.0195564 (PMC5898731; doi:10.1371/journal.pone.0195564)
Supplement: S1 Table — Knowledge about the self-limited disease course and subjective discomfort during the last common cold stratified by country and site (n = 2,204). (DOCX) [file pone.0195564.s001.docx]

**S1 Appendix.** **Knowledge about the self-limited disease course and subjective discomfort during the last common cold stratified by country and site (n=2,204).**

|  |  |  |  | **Knowledge of self-limitedness** | | | | |  | **Subjective discomfort** | | | | |
| --- | --- | --- | --- | --- | --- | --- | --- | --- | --- | --- | --- | --- | --- | --- |
| **Participating country** | **Site no.** | **Sample size** | **Res-ponse** | Self-limited condition: no | |  | Self-limited condition: yes | |  | Feel very poorly: no | |  | Feel very poorly: yes | |
|  |  | n |  | n | % |  | n | % |  | n | % |  | n | % |
| **Total** |  | **2,204** |  | **800** | **36.3** |  | **1,404** | **63.7** |  | **648** | **29.4** |  | **1,556** | **70.6** |
| **France** | 1 | 82 | 68.3 | 8 | 9.8 |  | 74 | 90.2 |  | 40 | 48.8 |  | 42 | 51.2 |
|  | 2 | 93 | 77.5 | 38 | 40.9 |  | 55 | 59.1 |  | 35 | 37.6 |  | 58 | 62.4 |
|  | 3 | 90 | 75.0 | 29 | 32.2 |  | 61 | 67.8 |  | 37 | 41.1 |  | 53 | 58.9 |
|  | Total | 265 | 73.6 | 75 | 28.3 |  | 190 | 71.7 |  | 112 | 42.3 |  | 153 | 57.7 |
| **Germany** | 1 | 74 | 61.7 | 31 | 41.9 |  | 43 | 58.1 |  | 13 | 17.6 |  | 61 | 82.4 |
|  | 2 | 87 | 72.5 | 24 | 27.6 |  | 63 | 72.4 |  | 33 | 37.9 |  | 54 | 62.1 |
|  | 3 | 131 | 109.2 | 33 | 25.2 |  | 98 | 74.8 |  | 33 | 25.2 |  | 98 | 74.8 |
|  | Total | 292 | 81.1 | 88 | 30.1 |  | 204 | 69.9 |  | 79 | 27.1 |  | 213 | 72.9 |
| **Italy** | 1 | 116 | 96.7 | 3 | 2.6 |  | 113 | 97.4 |  | 69 | 59.5 |  | 47 | 40.5 |
|  | 2 | 32 | 26.7 | 7 | 21.9 |  | 25 | 78.1 |  | 7 | 21.9 |  | 25 | 78.1 |
|  | Total | 148 | 61.7 | 10 | 6.8 |  | 138 | 93.2 |  | 76 | 51.4 |  | 72 | 48.6 |
| **Poland** | 1 | 96 | 80.0 | 64 | 66.7 |  | 32 | 33.3 |  | 10 | 10.4 |  | 86 | 89.6 |
|  | 2 | 99 | 82.5 | 54 | 54.5 |  | 45 | 45.5 |  | 9 | 9.1 |  | 90 | 90.9 |
|  | Total | 195 | 81.3 | 118 | 60.5 |  | 77 | 39.5 |  | 19 | 9.7 |  | 176 | 90.3 |
| **Turkey** | 1 | 108 | 90.0 | 52 | 48.1 |  | 56 | 51.9 |  | 79 | 73.1 |  | 29 | 26.9 |
|  | 2 | 109 | 90.8 | 46 | 42.2 |  | 63 | 57.8 |  | 19 | 17.4 |  | 90 | 82.6 |
|  | 3 | 107 | 89.2 | 56 | 52.3 |  | 51 | 47.7 |  | 30 | 28.0 |  | 77 | 72.0 |
|  | 4 | 104 | 86.7 | 53 | 51.0 |  | 51 | 49.0 |  | 22 | 21.2 |  | 82 | 78.8 |
|  | 5 | 110 | 91.7 | 77 | 70.0 |  | 33 | 30.0 |  | 10 | 9.1 |  | 100 | 90.9 |
|  | Total | 538 | 89.7 | 284 | 52.8 |  | 254 | 47.2 |  | 160 | 29.7 |  | 378 | 70.3 |
| **Austria** |  | 86 | 71.7 | 19 | 22.1 |  | 67 | 77.9 |  | 28 | 32.6 |  | 58 | 67.4 |
| **Finland** |  | 77 | 64.2 | 5 | 6.5 |  | 72 | 93.5 |  | 11 | 14.3 |  | 66 | 85.7 |
| **Israel** |  | 104 | 86.7 | 14 | 13.5 |  | 90 | 86.5 |  | 57 | 54.8 |  | 47 | 45.2 |
| **Macedonia** |  | 269 | *224.2** | 113 | 42.0 |  | 156 | 58.0 |  | 34 | 12.6 |  | 235 | 87.4 |
| **Slovenia** |  | 89 | 74.2 | 53 | 59.6 |  | 36 | 40.4 |  | 17 | 19.1 |  | 72 | 80.9 |
| **Spain** |  | 64 | 53.3 | 16 | 25.0 |  | 48 | 75.0 |  | 9 | 14.1 |  | 55 | 85.9 |
| **Sweden** |  | 77 | 64.2 | 5 | 6.5 |  | 72 | 93.5 |  | 46 | 59.7 |  | 31 | 40.3 |

*health-care center with unknown number of questionnaires; response could not be calculated.
